# A high-efficiency L-band coaxial three-period relativistic Cherenkov oscillator

**DOI:** 10.1038/s41598-019-47496-8

**Published:** 2019-08-22

**Authors:** Xing-Jun Ge, Peng Zhang, Chen-Yu Zhao, Zhi-Cheng Luo, Si-Yao Chen, Han-Wu Yang, Jun Zhang

**Affiliations:** College of Advanced Interdisciplinary Studies, National University of Defense Technology, Changsha, Hunan 410073 People’s Republic of China

**Keywords:** Electronic and spintronic devices, Plasma physics

## Abstract

Miniaturization is one of the important research directions of low frequency high power microwave sources. This paper presents a three-period coaxial slow-wave structure L-band high-power microwave source. Because the coaxial Quasi-TEM mode has no cut-off frequency, the radial size of the device can be reduced. At the same time, in order to reduce the transverse dimension, the coaxial extractor structure is introduced to realize the longitudinal mode selection and improve the conversion efficiency of the device. In simulation, the device obtains the microwave output with the central frequency of 1.53 GHz, the average power of 3.3 GW and the efficiency of 40%. By optimizing the scheme of electron beam collection, the phenomenon of pulse shortening is effectively suppressed. In the experiment, the device obtains the microwave output with the central frequency of 1.52 GHz, the average power of 3 GW, the efficiency of 33% and the pulse width of 40 ns.

## Introduction

High power microwave (HPM) source, as a research hotspot of relativistic vacuum electronic devices, has made great progress in the past decades^1–5^. It has a wide range of scientific and civil applications, such as high-power radar, plasma heating, particle accelerator and space propulsion^6–8^. Based on Cerenkov mechanism, relativistic backward oscillator (RBWOs) is an HPM generator with compact structure, large power, high efficiency, good stability and broad application prospect^9–13^. After decades of development, its band covers from L-band to Ka-band, and its output microwave power is 1–7 GW, its beam-wave conversion efficiency is 10–40%, and its repetition rate is 5–100 Hz^14–22^.

The L-band HPMs have important application prospects in civil and defense fields due to its advantages of a small free-space transmission loss and a strong diffraction, which attract the attention of scientific researchers. However, the biggest challenge in studying an L-band relativistic Cherenkov oscillator is to realize the miniaturization due to the size co effect. Consequently, the investigations on the compact low band relativistic Cherenkov oscillators with high power and high efficiency are research hotspots to the HPM field^15–17,21^.

In this paper, a high-efficiency L-band coaxial three-period relativistic Cherenkov oscillator is studied theoretically and experimentally. In order to reduce the size of the device, measurements in two aspects can be taken. One is to use the coaxial SWSs to excite the coaxial quasi-TEM mode, which has no cut-off frequency. The radial size of the device can be reduced obviously. The other is to design a coaxial extractor to achieve the longitudinal mode selection and reduce the axial length of the beam-wave interaction region. Based on this technical, which the longitudinal modes competition can be supressed and the beam-wave conversion efficiency can be improved. It is worth pointing out that the experimental results are consistent with the theoretical and simulation results.

## Device Design and Physical Analysis

**Figure 1:**
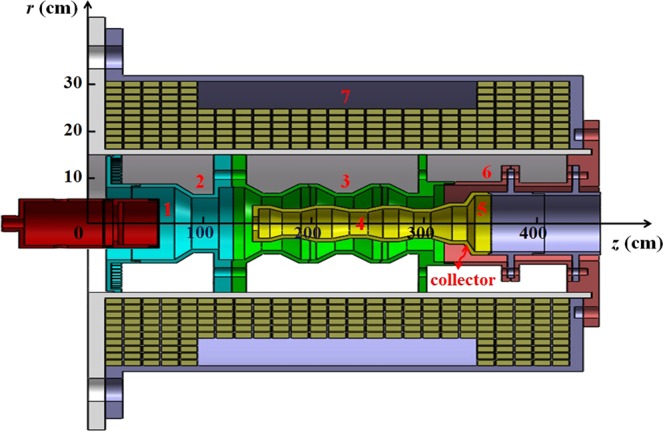
Schematic of the high-efficiency coaxial three-period relativistic Cherenkov oscillator: 1- cathode, 2-cut-off neck, 3- three-period outer SWSs, 4- three-period inner SWSs, 5-coaxial extractor, 6-output port, and 7-coil.

As shown in Fig. 1, the high-efficiency L-band relativistic Cherenkov oscillator consists of an anode, a circular cathode, cut-off neck, three-period coaxial SWSs, a coaxial extractor, an output port, and a coil. The coaxial SWSs have both inner and outer ripples, which can increase the coupling impedance and the temporal growth rate of the SWSs. Placed in the downstream of inner ripples is a coaxial extractor to increase reflection and improve efficiency. The coil is used to generate a guiding-magnetic field to maintain the electron beam stable transmission. The following focuses on the functions of the coaxial SWSs and the coaxial extractor.

### Analysis for functions of the coaxial SWSs

The coaxial SWSs are investigated systematically in our previous work^23^. Under the boundary conditions at the wall radii of the inner and outer SWSs and at the radii of the electron beam, we can obtain a homogeneous matrix equation by solving the Maxwell’s equations, the continuity equation and the motion equation with the field matching method. It is well known that the beam-wave interaction relies on the point multiplication between the electron beam density $\mathop{j}\limits^{\rightharpoonup }$ and the electric field $\mathop{E}\limits^{\rightharpoonup }$. The longitudinal electric field ${E}_{z}$ of the quasi-TEM and the TM modes are unequal to zero. Thus, the dispersion relations of the quasi-TEM and the TM modes arrest our attentions. Consequently, the dispersion curves obtained through the numerical calculation stand for the TM and the quasi-TEM modes.

**Figure 2:**
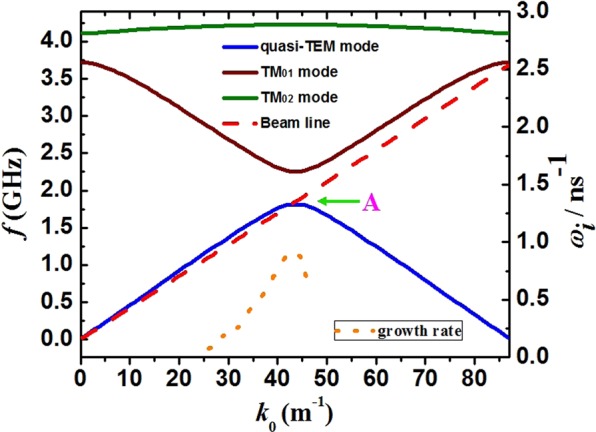
Dispersion curves of three modes, temporal growth rate and beam Doppler line.

Figure 2 shows the dispersion curves of coaxial SWS and the beam Doppler line with diode voltage of 600 kV. It can be seen that the beam Doppler line intersects with the quasi-TEM mode dispersion curve in the π-mode region. The coupling impedance and Q value of the π-mode are large, which are beneficial to improve efficiency. There are no intersection points between the electron beam line and other dispersion curves, which can avoid mode competitions.

The calculation results show that only quasi-TEM mode has a growth rate with the beam line, and the maximum value is near the intersection point. Moreover, the cut-off frequency of the quasi-TEM mode is equal to 0. It is means that the transversal dimension of the coaxial SWSs can be reduced remarkably, which is beneficial to the device miniaturization.

**Figure 3:**
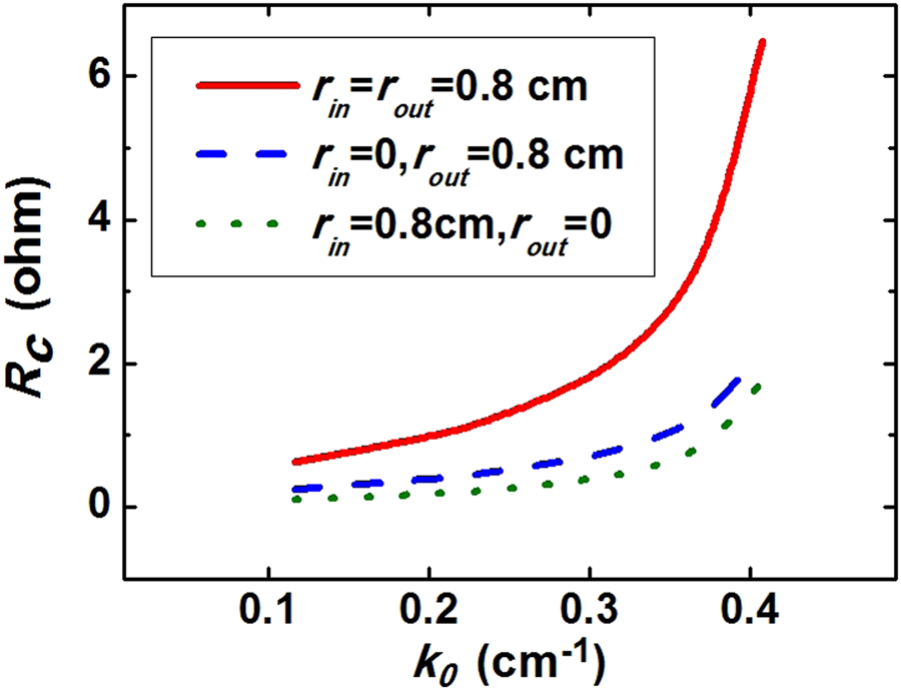
Coupling impedance of fundamental space harmonics in the SWSs with double ripples, outer ripple, and inner ripple. Here *r*_in_ and *r*_out_ represent inner ripple depth and outer ripple depth, respectively.

The coupling impedance of fundamental space harmonics in the SWSs with double ripples, outer ripple and inner ripple are obtained by numerical calculation, as shown in Fig. 3. The red solid curve stands for the coupling impedance of the SWSs with double ripples. It is clearly found that the SWSs with double ripples have the highest coupling impedance. The coupling impedances of the SWSs with outer ripple and inner ripple decrease in order. Obviously, the SWSs with double ripples can contribute to improving the beam-wave conversion efficiency. Naturally, the coaxial SWSs with double ripples are introduced to the L-band relativistic Cherenkov oscillator.

### Analysis for functions of the coaxial extractor

**Figure 4:**
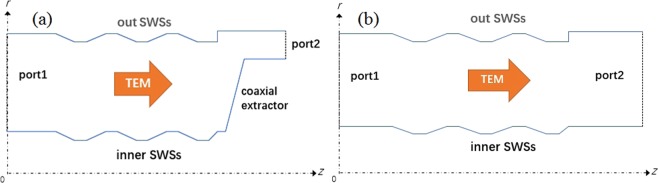
Schematic diagram of the (**a**) three-period SWSs with a coaxial extractor and (**b**) three-period SWSs.

In our previous work, the S-parameter method is employed to investigate the axial resonant characteristics of the finite-length coaxial SWSs^23^. To illustrate the characteristics of the coaxial extractor, the comparative investigations on the three-period coaxial SWSs with and without a coaxial extractor are conducted by the S-parameter method. In Fig. 4, the TEM mode is the exciter in two structures from port 1 to port 2, respectively.

Assume that there is a perfect match in the input and output sections of the two structures. So, we can use the S-parameter to analyze the longitudinal mode selection and define transmission coefficient T as

1 $$T(f)={S}_{out}^{+}/{S}_{in}^{+},$$

where ${S}_{out}^{+}$ and ${S}_{in}^{+}$ are the electromagnetic power fluxes for the direct waves through the input and the output cross-section of a structure, respectively.

**Figure 5:**
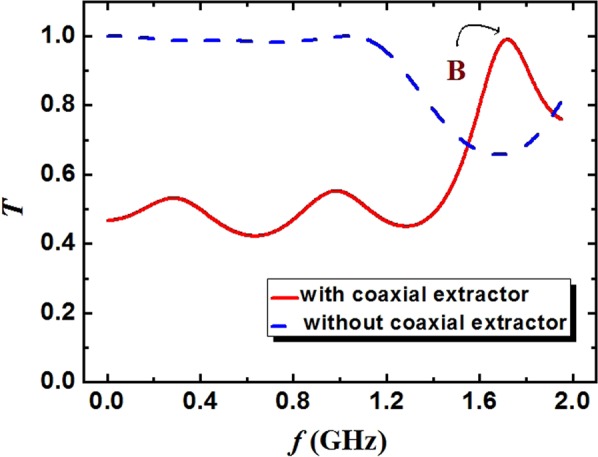
Transmission coefficients dependence on the frequency of the TEM mode in two structures.

The transmission coefficient varying the frequency in two structures is shown in Fig. 5. Each peak of the curve represents the longitudinal mode of the TEM mode. Compared with the transmission coefficient of three-period SWSs without the coaxial extractor (blue line), the transmission coefficient of the three-period SWSs with the coaxial extractor (red line) is obviously low in the range of 0–1.5 GHz. However, the resonance characteristic of former is significantly obvious than that of the latter. Particularly, the most obvious resonance point B occurs in the range of 1.5–2.0 GHz, which stands for the π -like mode and has a high transmission coefficient. Namely, the longitudinal mode (point B) can be excited and transmitted preferentially. Therefore, the introduction of the coaxial extractor can be beneficial to high efficiency and frequency stability.

## Results of Particle-in-Cell (PIC) Simulation

**Figure 6:**
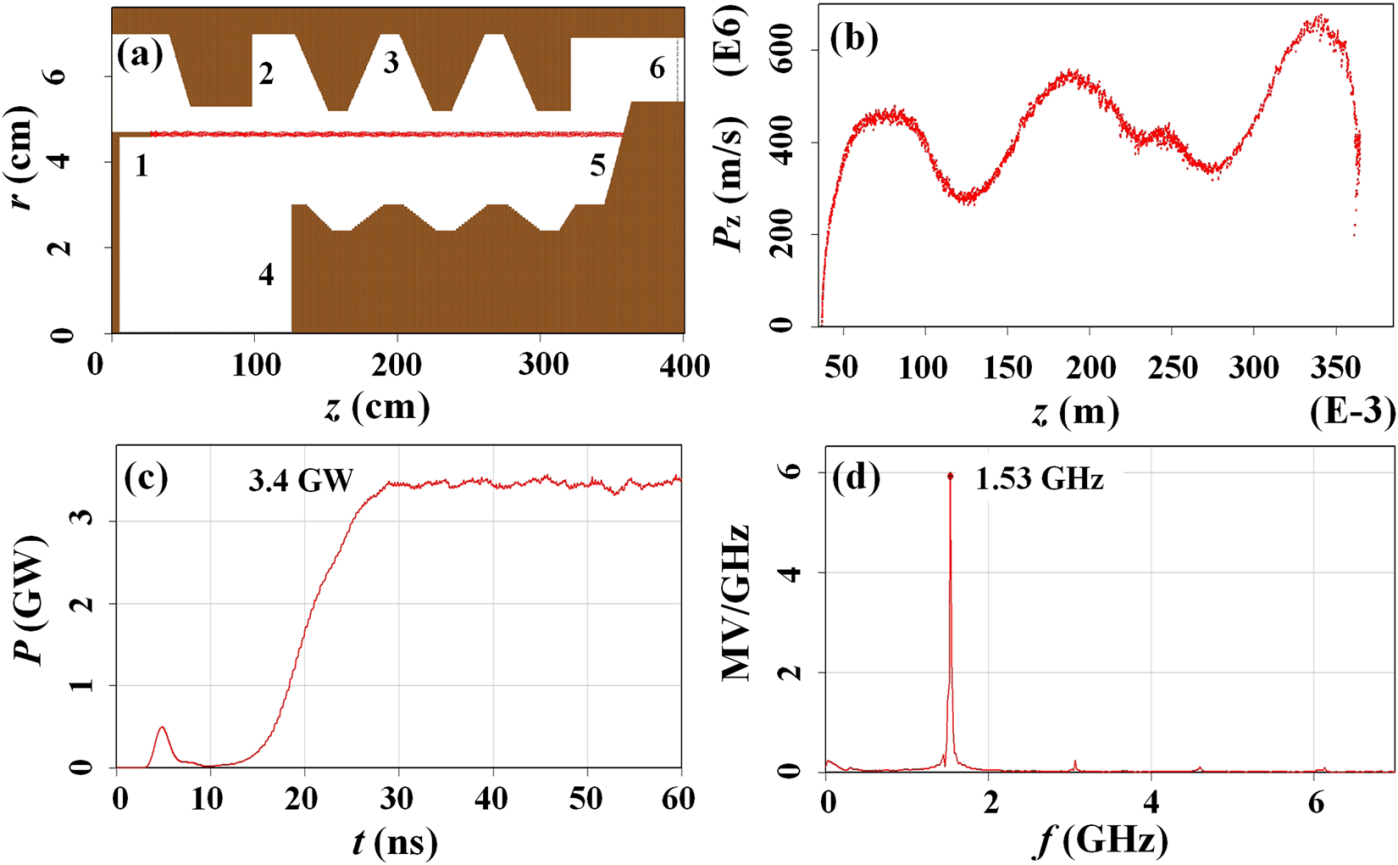
Simulation results of the L-band coaxial three-period relativistic Cherenkov oscillator: (**a**) the PIC simulation model (1-circular cathode, 2-cut-off neck, 3-outer SWSs, 4-inner SWSs, 5-coaxial extractor, 6-output waveguide), (**b**) the phase-space distribution of electron beam, (**c**) average power versus time, and (**d**) spectrum of output power.

To verify the correctness of the physical analysis with the high-efficiency L-band coaxial three-period relativistic Cherenkov oscillator operation, PIC simulations were carried out with the full electromagnetic code CHIPIC^24^. The PIC simulation model is illustrated in Fig. 6(a). In the model, the inner and outer conductors are connected with an optimized inductor, which ensures that the inner and outer conductors share the same potential. The working mechanism of the device will be briefly introduced as follows. The circular electrons emitted by the cathode with a knife-edge graphite emitter in the diode region.

The beam-wave interactions occur primarily in the coaxial SWSs region where electrons are fully bunched and transfer energy to the electromagnetic field. Finally, the increasing electromagnetic field induces a radio frequency voltage through the extractor gap, producing a TEM wave in the output waveguide around the coaxial extractor. In simulation, the diode voltage is 600 kV and the magnetic field is 1 T.

The typical physical images are shown in Fig. 6. Figure 6(a,b) give the real-space and phase-space distribution of the electron beam after microwave start-up. It can be seen that the velocity and density modulations are obtained when the electron beam enters the upstream SWSs region. The electrons bunch sufficiently in the third SWS, where energy is transmitted from the electrons to the electromagnetic field. Naturally, the well-bunched electrons deliver energy continuously to the electromagnetic field, and then the velocities of the electrons drop obviously. Consequently, the exhausted electrons dump on the coaxial extractor and disappear in the phase-space distribution.

By calculating the integral of the Poynting flux on the cross section of the output waveguide, the change of the microwave output power with time is shown in Fig. 6(c). As can be seen, HPM vibrates from 10 ns and saturates at 29 ns. Obviously, the average power after saturation is about 3.3 GW, and the corresponding beam-wave interaction efficiency is about 40%. Meanwhile, the output microwave power spectrum obtained by the fast Fourier transform (FFT) of the saturated microwave power is shown in Fig. 6(d). The output microwave spectrum is relatively pure with a central frequency of 1.53 GHz, which effectively inhibits frequency doubling and high-order mode. In other word, it shows the good propagation of the electron beam and the effective interaction between the electron and the electromagnetic field.

## Experimental Results

### Experimental setup and HPM measurement

**Figure 7:**
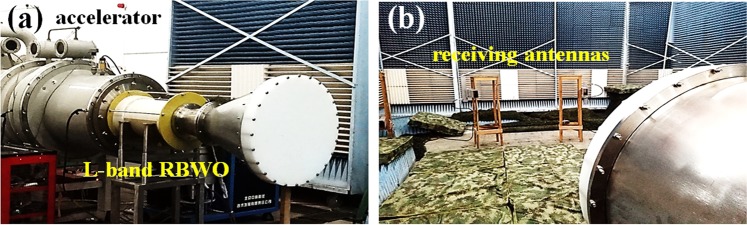
Experimental layout of the (**a**) accelerator and L-band Cherenkov oscillator and (**b**) measuring system.

An L-band coaxial three-period relativistic Cherenkov oscillator is operated on the TORCH-01 accelerator, as shown in Fig. 7. The TORCH-01 accelerator is capable of providing an electrical power up to 10 GW with a full width at half maximum (FWHM) pulse duration of about 55 ns. The guiding-magnetic field and its profile are measured by a Tesla-meter based on the Hall-effect. The diode voltage and the beam current are measured by a capacitance voltage divider and a Rogovsky coil, respectively.

Microwave measurement system consists of receivers, microwave cables, attenuators, crystal detectors, etc. In Fig. 7, the radiated microwave from the horn antenna is measured by two receivers (one is fixed as a reference and the other one is shifted). The distance between the phase centre of the horn antenna and the receiver is 5 m. Microwave signal is measured by oscilloscope through 40 m microwave cable, attenuators, and a crystal detector. All detectors are well covered by microwave absorbing material to prevent electromagnetic noises. The oscilloscope is placed in a shielding room. Consequently, the microwave power is obtained by integrating over the radiation pattern. The microwave frequency is obtained through FFT of the microwave signal in the oscilloscope without the crystal detector.

### Primary experimental results

**Figure 8:**
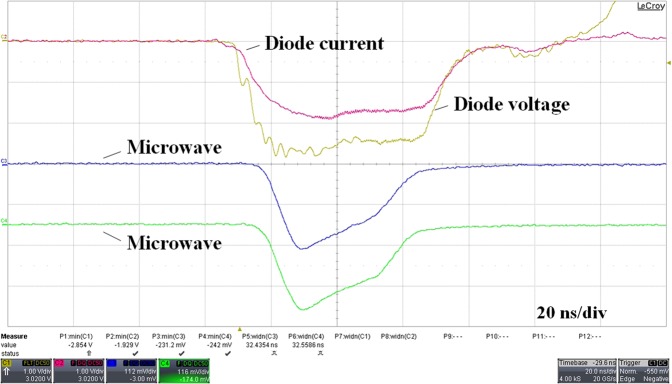
Typical pulse shorting waveforms in the initial experiment.

Under the typical parameters of diode voltage 600 kV, beam current 15.5 kA, and guild-magnetic field 1 T, the initial experimental result is shown in Fig. 8. It is found that there occurs significant pulse shortening phenomenon in Fig. 8.

**Figure 9:**
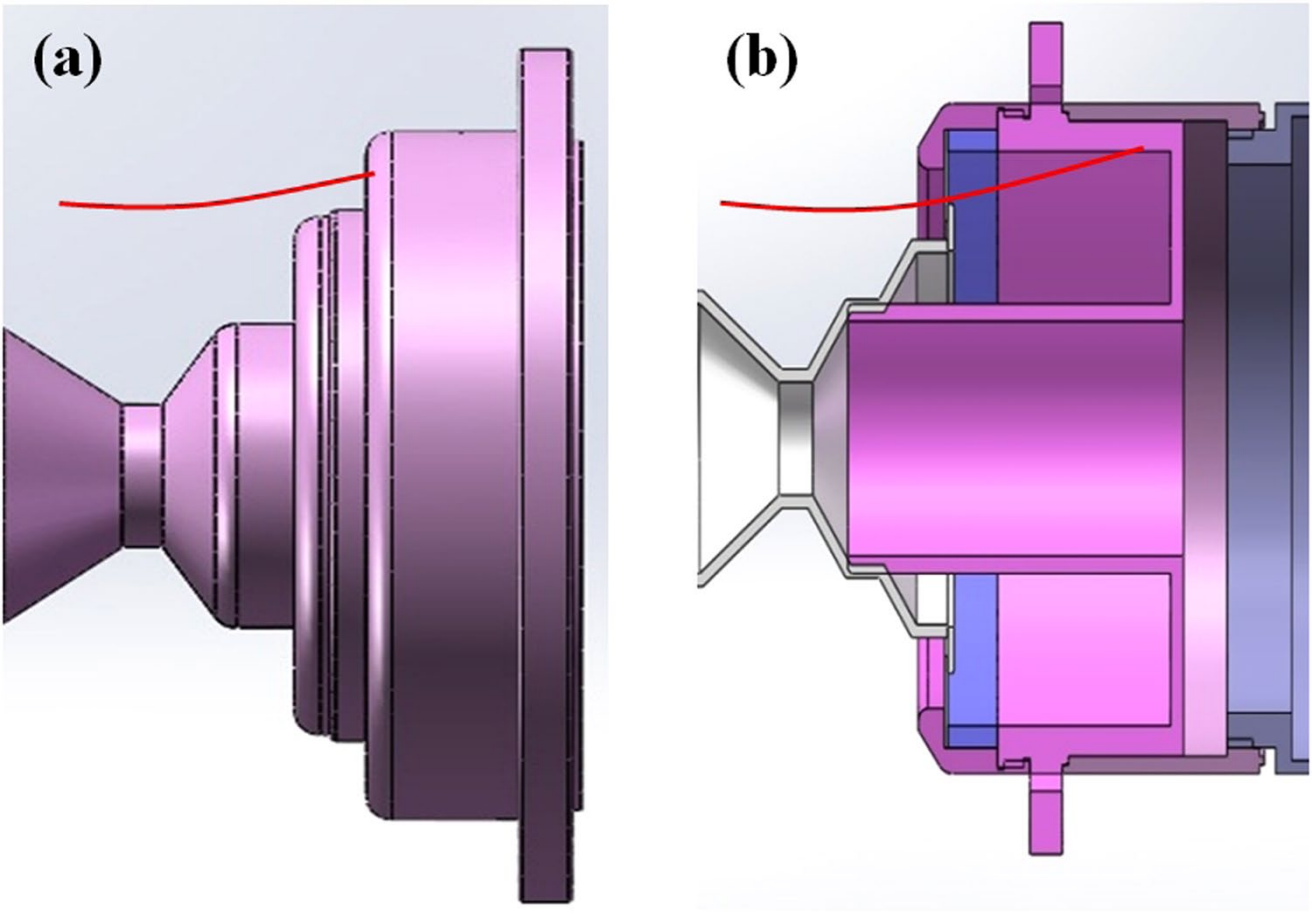
Electron beam is collected by (**a**) the left end of the coaxial extraction and (**b**) the inside of the coaxial extractor.

**Figure 10:**
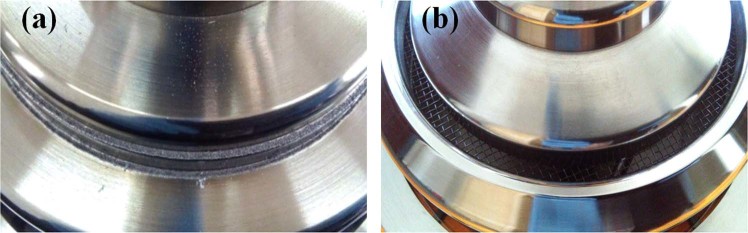
Pictures of the left end of the coaxial extraction after the experiment: (**a**) electron beam directly bombards and (**b**) electron beam transmits through an annular groove.

Through parameter adjustment and comparative analysis, it is believed that the reason may be the influence of electron beam collection. In the original design, the electron beam directly bombards the left end of coaxial extraction, as shown in Fig. 9(a). The plasma generated by electron bombardment on the coaxial extraction may flow back along the magnetic line to the slow-wave region, which affects beam-wave interaction. After the experiment, there are obvious bombardment marks and metal powder on the coaxial extraction end face, as shown in Fig. 10(a).

**Figure 11:**
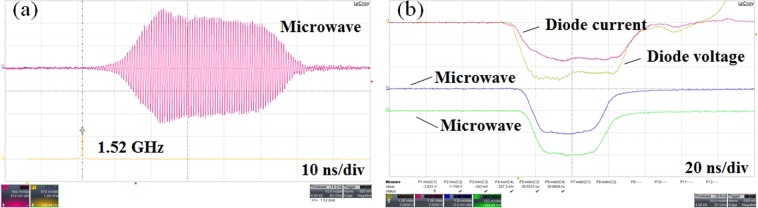
Typical measured waveforms of the (**a**) radiated microwave and its spectrum and (**b**) diode voltage, current and microwave.

Some measures are taken to solve the problem of electron beam collection, as shown in Fig. 9(b). Electron beam transmits through an annular groove and bombard the inside of the coaxial extractor. The molybdenum mesh in the annular groove is introduced to isolate microwave. After structural optimization, the beam-wave interaction efficiency reduces from 40% to 36% in simulation. Figure 10(b) gives a picture of the molybdenum net collector after the experiment. It can be seen that there are no obvious bombardment marks. In the experiment, the microwave waveform is significantly improved, and the pulse shortening is effectively suppressed, as shown in Fig. 11(b).

With the same operating condition, the typical improved experimental results are shown in Fig. 11. Figure 11(a) shows the output microwave waveform and FFT results. It is worth noting that the spectrum of microwave output is very pure, with a centre frequency of 1.52 GHz. As shown in Fig. 11(b), the output microwave varies with time, and the output microwave with power of 3 GW and pulse width of 40 ns is obtained. Compared with the input electric power, the beam-wave interaction efficiency is about 33%. However, the experimental efficiency is slightly lower than the simulation results (36%). This may be because the experimental beam based on the emission of exploded electrons is not as good as in simulation. In addition, processing and assembly tolerances also affect the efficiency of the device.

**Figure 12:**
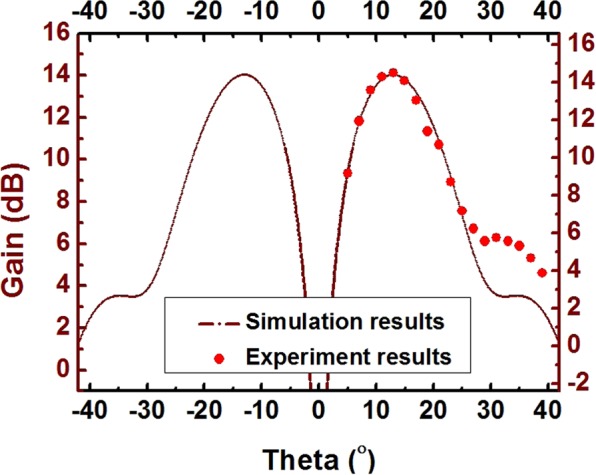
The measured microwave radiation pattern and the theoretical pattern of the symmetric TM_01_ mode.

In simulation, the operation mode (quasi-TEM mode) propagating along the coaxial waveguide and is converted to the TM_01_ mode by mode converter. The TM_01_ mode is then transmitted through the horn antenna. The measured pattern of microwave radiation (as shown in Fig. 12) agrees well with the theoretical mode of the symmetric TM_01_ mode, indicating that the dominant mode of the device is the quasi-TEM mode.

## Conclusion

To sum up, we demonstrate theoretical, simulation and experimental investigations concerning a high-efficiency L-band coaxial three-period relativistic Cherenkov oscillator, which can produce HPM radiations with the peak power of 3 GW, the centre frequency of 1.52 GHz, the pulse duration of about 40 ns, and beam-wave conversion efficiency of 33%. It is particularly worth pointing out that the introductions of coaxial three-period SWSs and a coaxial extractor contribute to the compact structure and the high beam-wave conversion efficiency.

Despite some good achievements that we have made, there do exist some features of the device which should be enhanced in order to chase the practical applications in HPM systems: (i) The high beam-wave conversion efficiency should be further improved to reduce the input electric power, which is beneficial to decreases of the risk of insulation and stabilities of the operation. (ii) The pulse shortening mechanisms in the device should be investigated to prolong the pulse duration. (iii) The pulsed magnetic field should be studied to reduce the volume and weight of the excitation system.
